# Notes on Current Neurology

**Published:** 1907-09-28

**Authors:** Purves Stewart


### Notes on Current Neurology.

By PURVES STEWART, M.D., F.R.C.P.

### NEUROSES AND PSYCHO-NEUROSES.

The boundary between organic diseases and so-called "functional" diseases of the nervous system is not so definite as might at first sight be supposed. It is, in fact, a zone rather than a line of demarcation. A neurosis is commonly defined as a nervous affection devoid of anatomical changes. Such a definition, however, is simply a confession of our etiological ignorance, for it is impossible to conceive a disease devoid of a lesion. The lesions underlying the neuroses may not be visible microscopically; they may be of a chemical, bio-chemical, or molecular nature; as a rule they are less severe than in ordinary so-called organic diseases, but they are none the less real. In functional diseases the changes are not irreparable, unlike diseases due to gross anatomical lesions, which always leave some trace of the old trouble behind.

With advancing knowledge the number of diseases included within the category of neuroses has steadily diminished. Thus, for example, tabes, exophthalmic goitre, and chorea, which were formerly classed as neuroses, have been transferred to the class of organic affections; whilst there is little doubt that epilepsy and paralysis agitans, in view not only of their clinical phenomena, but also of their steadily progressive nature, should, although their specific pathogeny is as yet undiscovered, be separated from the group of neuroses proper. But, as we have seen, in the neuroses it is not the absence of lesions, but the absence of recognised lesions, which constitutes their distinguishing feature. Taking this as our standard, even hypochondriasis, which by many authorities is considered as a psycho-neurosis, must be excluded from the group of neuroses proper. As Raymond has recently emphasised in an interesting series of lectures on this subject,* we have to recognise two varieties of hypochondriasis. In the one, the hypochondriasis is symptomatic of some underlying morbid sensation due to actual structural disease; it is, in fact, merely an exaggeration of a real sensation. In the other variety the hypochondriasis is independent of any discoverable structural change. But in both varieties the essential point is the mental condition of the hypochondriac patient, consisting in a misrepresentation of his sensations; hypochondriasis, in fact, is a symptom of mental affection.

Passing now to the neuroses properly so-called, three conditions are included under this category—namely, neurasthenia, psychasthenia, and hysteria. I will endeavour to summarise Raymond's recent pronouncements on the subject.

The distinction between neurasthenia and psychasthenia is an important one. Neurasthenia proper, like dyspepsia, is a syndrome rather than an actual disease. Its outstanding features consist of "irritable feebleness," together with general depression of all the functions of the nervous system. Its psychical symptoms are simple and elementary. The general nervous depression manifests itself in such phenomena as instability, rapid fatigue, restlessness, undue emotion and downheartedness. Neurasthenia is a comparatively simple, acquired affection, which with proper treatment is usually curable. It is not a disease *per se*, but a syndrome which can be produced by numerous depressing causes, organic or otherwise. Psychasthenia, on the other hand, is a more serious affair, in which the mental phenomena overshadow the physical; its outstanding features are psychical, chief amongst which are aboulia, or want of initiative power, various persistent obsessions and "phobiæ," fixed ideas, systematised tics, and so on. Another point of contrast is that neurasthenia is usually a disease of adult life, the result of some accidental cause, physical or psychical, which enfeebles the nervous system. Before the onset of neurasthenic symptoms the patient is free from nervous taint, and after the neurasthenia has passed off he is normal again. Psychasthenia, on the contrary, is the climax of a deep-seated hereditary or congenital predisposition, indications of which are evident in early life, childhood or adolescence, and gradually attain their full development without apparent exciting cause. In short, psychasthenia is a weed which blossoms only in the rank soil of a degenerate nervous constitution. The future psychasthenic, before attaining the full development of his disease, is a neuropathic, listless individual, showing various abnormalities in the form of aboulia, excessive scruples, obsessions, perhaps one of the many forms of tic, and so on. Unlike the neurasthenic patient, who is eminently curable, the psychasthenic individual is never completely cured; he retains certain permanent psychasthenic stigmata, to which we shall return presently.

Hysteria, psychasthenia and neurasthenia occur as fairly distinct clinical entities, but there are many mixed and transitional forms. Thus, especially in incomplete forms of functional disease, it is sometimes difficult, in practice, to classify accurately an individual case.

### Treatment of Psycho-neuroses.

Certain general principles of treatment are applicable to all the psycho-neuroses, and have been well summarised by Raymond. The three outstanding symptoms which call for treatment are general nervous irritability, insomnia, and malnutrition. These three are almost constantly present, the one reacting on the other, constituting a vicious circle, a circle which we must attack simultaneously from all sides.

The first essential is to remove the patient from his home surroundings. This is a *sine qua non* for a successful result. In the early stages of treatment, at least, the isolation should be absolute. All outside sources of excitement and distraction are thus excluded, and the patient can devote his entire attention to his cure. Complete confinement to bed is also

* L'Encephale, 1907.

essential at the outset, but Raymond does not believe it necessary to keep the patient in bed throughout the cure, though he insists on his always going to bed early and rising late.

Hypnotics, when required, must be employed without hesitation and in sufficient doses. Raymond does not specially recommend bromides for this purpose, since they tend to depress the patient and sometimes induce gastric disorder. But if bromide or any other sedative drug be deemed necessary, there should be frequent intermissions in their administration, giving them, say, on alternate nights, so as to avoid cumulative effects. What keeps some patients awake is the mere apprehension of insomnia, and it is often convenient to give the patient a sedative mixture to keep by him, telling him not to take it unless he finds it necessary, say, unless he lies awake for two or three hours after retiring to rest. Often the knowledge that a sleeping-draught is available relieves his apprehension, and he falls asleep without taking it.

The general malnutrition is treated on the lines with which Weir-Mitchell has made us familiar. Special diet is necessary, commencing with milk alone, and rapidly adding other articles until the patient is taking a super-normal amount of food. The patient must be weighed regularly, and as his weight goes up he should be told of it, so as to encourage him and stimulate him to further alimentary feats.

The mental anxiety and depression which is so frequently present in these cases is best treated, according to Raymond, by laudanum, in alternately increasing and decreasing doses. A convenient daily quantity to begin with is 20 minims divided into two doses, one half taken in the forenoon and the other half some three hours after the evening meal. The daily amount is increased by 2 or 4 minims at a time. Sometimes a maximum of 100 minims is reached, but usually from 60 to 80 minims per diem is enough. Aperients must be employed to counteract the tendency to constipation which results from the opium.

Moral treatment.—"psycho-therapeutics"—in such cases is of the highest importance. Tact and *savoir faire* on the part of the physician are indispensable. He must try at the outset to gain the patient's confidence, and must avoid the common mistake of letting the patient have the impression that he (the patient) is considered as a *malade imaginaire*. The physician must impress on the patient that the disease, whilst real, is a curable one. He must try to restore the patient's self-confidence, and encourage him by pointing out each improvement that occurs as the case progresses. Hypnotism is to be employed only in exceptional cases, and then only for a short period.

Amongst the accessory therapeutic measures must be mentioned hydro-therapeutical and electrical procedures. In conditions of mental depression cold douches or tepid baths of short duration are specially indicated, whereas excited patients do best with prolonged warm baths, tepid douches, and wet-packs. A warm bath for a half to three-quarters of an hour just before bedtime often acts as an excellent hypnotic. Amongst electrical methods, static electricity is the best. Massage and systematic exercises are also of great assistance in combating the malnutrition and favouring assimilation of the abundant diet. Tonics are also of use, and we must not omit to treat with care any co-existing symptoms which may be present along with the nervous malady.

